# Risk Factors for the Efficiency of Artificial Insemination in Dairy Cows and Economic Impact of Failure of First Service Insemination in and around Haramaya Town, Oromia Region, Eastern Ethiopia

**DOI:** 10.1155/2021/6622487

**Published:** 2021-05-21

**Authors:** Muhammed Hamid, Sadam Abduraman, Belege Tadesse

**Affiliations:** ^1^Samara University, College of Veterinary Medicine, PO. Box. 132, Samara, Ethiopia; ^2^Wollo University, School of Veterinary Medicine, PO. Box 1145, Dessie, Ethiopia

## Abstract

A cross-sectional study was conducted from November 2019 to May 2020 in and around Haramaya Town to study the risk factors of artificial insemination (AI) in dairy cattle and evaluate the economic impact of failure of first service AI. A questionnaire survey and field follow-up were employed for collecting data from cattle owners and artificial insemination technicians (AITs) who were selected purposively. Out of the 221 inseminated cows and heifers, the overall conception rate was 60.2% (*n* = 133). The conception rate was statistically different between breed (*P*=0.019) and insemination time (*P*=0.049). From a total of 133 conceived cows and heifers, the conception rate was 68 (53.54%) in local breeds and 65 (69.15%) in cross breeds. Parity, age of cows, inseminator experience, and body condition of cows did not create a significant difference in conception rate (*P* > 0.05). Failure to conceive at their first AI results in an extra cost of 440 ETB per day until conception. Therefore, to increase the conception rate, dairy cows should be inseminated early when they show signs of estrous; the owners of dairy cows should be trained on how to detect estrous signs in dairy cows and AI technicians should also take training in order to improve their skills.

## 1. Introduction

Ethiopia is the largest livestock population in Africa, estimated to be 59.5 million cattle, 60.9 million sheep and goats, about 1.2 million camels, and 59.5 million chickens [1]. “Agriculture,” mainly crop and livestock production, is the mainstay of the Ethiopian economy employing approximately 85% of the total human population. Livestock production accounts for approximately 35–49% of the total agricultural GDP and 16 to 17% of national foreign currency earnings [2].

According to the report of CSA [1], from total cattle population of the country 44.5% and 55.5% are male and females, respectively. Based on breed, 98.2%, 1.62, and 0.18 were local breeds, cross breeds, and exotic breeds, respectively. The low genetic potential and poor technical knowledge of dairy owners limit the incomes of dairy producers [3].

The low productivity of the indigenous cattle and current rapid growth of human population make it difficult to meet the protein requirements of the population, [4]. Cattle production system in Ethiopia is mainly smallholder subsistence farming, with animals having multipurpose use, and such that no specialized and systematic breeding is used [5]. Artificial insemination has been considered as a promising tool to improve genetic potential of dairy animals; yet, many farmers at field conditions are unaware of the technology with huge regional variations in terms of knowledge level and adoption of this promising technology [6].

Artificial insemination has been defined as a process by which sperm is collected from the male, processed, stored, and artificially introduced into the female reproductive tract for the purpose of conception [7]. Semen is collected from the bull, deep-frozen, and stored in a container with liquid nitrogen at a temperature of minus 196 degrees Centigrade and made for use. Artificial insemination has become one of the most important techniques ever devised for the genetic improvement of farm animals. It has been widely used for breeding of dairy cattle as the most valuable management practice available to the cattle producer and has made bulls of high genetic merit available to all [7, 8].

Ethiopia uses AI service to improve productive efficiency over the last 30 years and increase the economic gain from the dairy sector [9]. However the efficiency of the AI service in the country is of very low level due to infrastructure, managerial, and financial constraints as well as poor heat detection and improper timing of insemination [10]. Cattle breeding is mostly uncontrolled in Ethiopia making genetic improvement difficult and appropriate bull selection criteria have not yet been established, applied, and controlled [11].

Although artificial insemination, the most commonly used and valuable biotechnology [7], has been in operation in Ethiopia for over 30 years, the service is still weak and even declining and the efficiency is also low [12]. Reproductive problems related to crossbreed dairy cows under farmers' conditions are immense [13]. It is widely believed that the AI service in the country has not been successful to improve reproductive performance of dairy industry [14]. The problem is more aggravated by lack of recording system, estrous detection problems, wrong selection procedures, and poor management of AI bulls and skills of inseminators [15]. The efficiency, risk factors of AI in dairy cows, and economic impact of failure of first service AI have not been well documented in Harar. Therefore, this study has been conducted with the following objectives:

- To determine the efficiency of AI service in and around Haramaya Town
- To identify risk factors that influence the efficiency of AI in the area
- To evaluate the economic impact of failure of first service AI

## 2. Materials and Methods

### 2.1. Description of the Study Area

**Figure 1:**
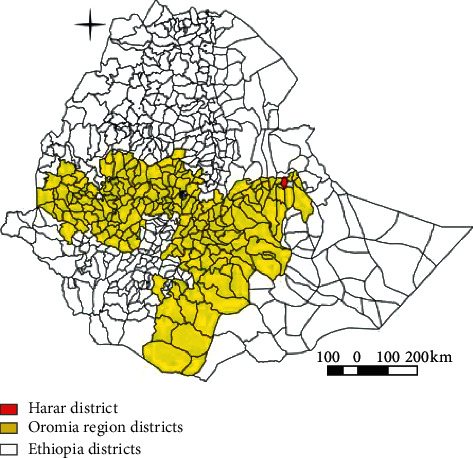
Map of study site (produced using QGIS 2.18.28).

The study was conducted from November 2019 to May 2020 in and around Haramaya Town, Eastern Hararghe zone, Oromia Region (Figure 1). Haramaya Town was located at 478 km to the Eastern of Addis Ababa. According to the information gained from Agricultural Office of the District, the agroclimate condition of the area falls within tropical subhumid climate as the area has 3 to 4 humid months. The altitude ranges from 1800 to 2345 meters above sea level and their longitude and latitude are 41°58′28.02″'-42°8′10.26″E and 9°23′12.27'–9°31′9.85″ N, respectively. The town has an average rain fall of 800.9 mm and average temperature of 17.04 °c. The main rainfall season for the town is from June to September and the dry season from December to April and their humidity was 57.83% [16].

### 2.2. Study Design

A cross-sectional study was conducted to determine the risk factors of efficiency of AI service through regular visit of selected dairy cattle breeder and AI technician from November 2019 to May 2020 in and around Haramaya Town, Oromia region. During the study period, special attention has been given to the AI service activities in the study areas.

### 2.3. The Study Population

The study populations were artificially inseminated cows kept under both extensive and intensive production system in and around Haramaya Town.

### 2.4. Method of Data Collection and Sample Size

Structured questioner survey was prepared and administered directly to the owner of the dairy cows. During the interview, the respondents included in the study were briefed about the objective of the study before presenting the actual questions. The questioners include address of owner, breed of animal, parity, body condition score, time of insemination, inseminators, managemental factors, and reproductive health problems. In addition to the questionnaire interview, field follow-up of inseminated cows was conducted to determine the efficiency of AI. Pregnancy diagnoses by rectal palpation have been done after two months according to Robert [17] and Arthur [18]. The time of insemination starting from observation of estrus signs (i.e., standing to be mounted, mounting other cows, swelling and reddening of the vulva, bellowing, restlessness, and trailing) has been classified into three including those inseminated within 6, between 6 and 11, and after 11 hours. The cows were observed frequently for estrus signs by the keepers and the keepers call the inseminators. Three inseminators who were involved in AI practice were considered based on their year of experience.

The sample size was determined by the availability of artificially inseminated cows in the study town. A total of 221 inseminated cows and heifers done by three inseminators were included in the study. Cows and heifers that were inseminated by AI were selected purposively from dairy cows in and around Haramaya Town.

The pregnancy rate was estimated by dividing the conceived cows by the cows that were inseminated.

### 2.5. Evaluation of Economic Impact of Failure of First Service Insemination

The economic impacts associated with the failure of first service conception by AI include the extra costs of AI until conception for both cows that have conceived at first AI and that have not conceived and management procedures for cows that failed to conceive at their first AI, incurred due to a higher number of days open. The extra costs include cost of extra feed fed in additional days + value of extra labor used for management of cows + value of milk loss due to the larger number of days open [19].

### 2.6. Data Analysis

All data were entered into Microsoft Excel spread sheet 2010, coded and analyzed using STATA version 13 statistical package. Descriptive statistics was used to describe the pregnancy rate and economic impact of failure of first service AI. Chi-square and multiple logistic regressions were used to check for any association between different risk factors and pregnancy rate. In all the analyses, confidence level was held at 95% and *P* value less than 0.05 was considered as significant.

## 3. Results

### 3.1. Risk Factors and Efficiency of AI

Out of the 221 dairy cows included in the study, 127 and 94 were local and crossbreed cows and heifers respectively. The overall efficiency of AI was 60.2% (*n* = 133) out of the 221 dairy cows and heifers included in the study. Breed-wise pregnancy rate was 53.5% (*n* = 68) in local breeds and 69.1% (*n* = 65) in cross breed cows and heifers. The pregnancy rate was significantly higher in cross breeds (*P*=0.019) and in within 6 h of insemination after the start of estrus signs (*P*=0.049), whereas the pregnancy rate has no significant difference between age, parity, body condition, and inseminators experience (Table 1).

### 3.2. Association of Efficiency of AI with Different Risk Factors

The odds of occurrence of pregnancy was significantly higher in cross breed cows and heifers as compared to local breeds (OR = 2.25 (1.2–4.2); *P*=0.011). Based on insemination time odds of occurrence of pregnancy was significantly lower in between six and eleven hour insemination as compared to the within six-hour insemination (OR = 0.38 (0.21–0.71); *P*=0.002), whereas there is no significant difference between age, parity, BCS and inseminators experience (Table 2).

### 3.3. Economic Impact of Failure of First Service Insemination

The cost of single insemination and pregnancy diagnosis (PD) was seven and 24 Ethiopian birr (ETB) respectively. In cows that failed to conceive at their first service AI, an additional expense of 440 ETB was incurred due to nutrition, milk loss, and labor per day until conception (Table 3).

## 4. Discussion

In the current study, the overall conception rate was 60.2%, whereas it was 68 (53.54%) in local breeds and 65 (69.15%) in cross breeds. Higher conception rate was found in cross breed as compared to local breed cows. This variation in conception between the two breeds could be due to genotype, heat detection accuracy, and farmers' biasedness to manage crossbred better than the local cattle. This finding is in agreement with that of Yeshitila et al. [20] who reported 75.5 and 72.9% in cross breed and local breed, respectively, around Kombolcha town. Other possible reasons for the lower proportion of indigenous cow conceiving at first insemination are that a Zebu cow does not exhibit overt estrus signs like crossbred cows [21,22]. Estrus manifestations have been known to be short, erratic, and mostly less evident or silent heat further requiring a meticulous observation and timely insemination to result in successful pregnancy [23].

The conception rate of cows in the present study were 11(57.9%) in cows with age of <3 years, 75(64.1%) in cows with age of 3–6 years, and 36 (60%) in cows with age of above 6–9 years. These findings were lower than those of Howlader [24] who reported 71.93% of dairy cows in the age of <3 years, 85.49% in the age of 4.6–6 years, and 74.52% in the age of >6 years, but higher than Alem and Sarader [25] who reported 33.33%, 38.5%, and 29.8% efficiency in dairy cows of <3 years, 4.6–6 years, and >6 years, respectively. It is also lower than the report of Yeshitila et al. [20] in Kombolcha town.

Regarding the time of insemination, in the present study a higher conception rate was observed in cows inseminated within six hours of showing estrous signs 84 (67.2%). This finding was slightly higher than the 65% conception reported by Howlader [24] and Yeshitila et al. [20]. However, Howlader [24] reported a higher conception rate in cows that were inseminated in the middle of estrus. The difference may be due to difference in the management of the semen and difference in the ability of the inseminator to correctly inseminate. Cows should be inseminated within six hours of heat to increase the chance of conception because late insemination may affect the conception rates [25, 26].

Based on parity, the conception rate of cows was 75 (59.1%) in multiparous cows and 58 (61.7%) in primiparous cows in the current study. These results were in agreement with those in the report of Alem and Sarader [25] which were 60.00% in primiparous cows and 66.7% in multiparous cows, but lower than the report of Yeshitila et al. [20]. In the current study, there is no much difference in conception rate between parity, whereas, in a study reported by Alexandra [27], conception rate was higher in multiparous cows. The variation in conception rate among parity may be due to the changes in managerial systems and environmental conditions among parities. According to Lucy et al. [28], negative energy balance causes a delay in interval to first ovulation and a delay in interval to first estrus.

In the current study, the conception rate was 55.2% and 66.7% in medium and moderate conditioned cows. This finding was lower than the report of Alam and Sarader [25] and Yesitila et al. [20]. The difference may be due to difference in the estrous detection ability of owner and AI technicians and time of insemination. This variation could be that cows with condition loss may not be ready to manifest the estrous signs early [29, 30].

Even if there was no significant difference in conception rate among the three inseminators, inseminators having significant amount of experience have high conception rate (65.9).

In the current study, failure to conceive at their first AI results in an extra cost of 440 ETB per day until conception. In addition to this a cost of 31 ETB was incurred per single insemination and PD. This is a huge cost as we compare with cows that conceive at their first service AI. According to Kim and Jeon (2019) an additional expense of $567.00 was incurred for the management procedures required to achieve conception. Another previous study showed when more than three inseminations per conception were needed for the cow to conceive, the profit of the owner was decreased by >$205/year per cow [31]. It is difficult to compare the economic loss between the current study and other previous studies, Sheldon et al. [32], directly because of different study design and values for the animals and factors considered. However, it is clear that when the numbers of services per conception increase, the economic loss becomes greater. In general, the amount of the economic loss may differ depending on the AI efficiency and the size of other expenses associated with management on dairy farms [33].

## 5. Conclusions and Recommendation

The present study indicates that the conception rate of cows by AI was moderate. The conception rate varies with the breeds of cows and time of insemination. The conception rate was high in cross breed cows and heifers and in within six hours of insemination after the cow starts to show the sign of heat. This study showed that delayed time of insemination is one of the important factors that decrease efficiency of AI in dairy cows. In cows that failed to conceive at their first AI, the owners fall in extra costs and losses due to large number of days open.

Therefore, based on the above conclusions, the following recommendations are forwarded:

- Dairy cows should be inseminated within the first six hours of showing estrous signs to achieve a good conception rate
- The owners of dairy cows should be trained on how to detect estrous signs in dairy cows
- The government should prepare trainings to AI technicians in order to improve their skills and enhance the efficiency of AI
- Awareness of the importance of FSC and strategies should be adopted to improve FSC in dairy herds

## Figures and Tables

**Table 1:** Efficiency of AI related to different factors.

Variables		Frequency	Pregnant (%)	Chi-square	*P* value
Age in years	≤3	19	11 (57.9)	3.52	0.318
	>3–6	117	75 (64.1)		
	>6–9	60	36 (60.0)		
	>9	25	11 (44.0)		

Breed of cows	Local	127	68 (53.5)	5.50	0.0.019
	Cross	94	65 (69.1)		
	Overall	221	133 (60.2)		

Insemination time	≤6 h	125	84 (67.2)	6.09	0.049
	>6–11 h	89	45 (50.6)		
	>11 h	7	4 (57.1)		

Parity	Primiparous	94	58 (61.7)	0.12	0.691
	Multiparous	127	75 (59.1)		

BCS	Medium	125	69 (55.2)	2.98	0.084
	Moderate	96	64 (66.7)		

Inseminator experience	<5 years of experience	101	61 (60.4)	1.02	0.602
	5–8 years of experience	76	43 (56.6)		
	>8 years of experience	44	29 (65.9)		

Overall	Overall	221	133 (60.2)		

**Table 2:** Multiple logistic regression result showing the association of pregnancy rates with different factors.

Variable		OR (CI)	*P* value
Breed	Local	Ref.	—
	Cross	2.25 (1.2–4.2)	0.011

Age in years	≤3	Ref.	—
	>3–6	1.35 (0.44–4.14)	0.604
	>6–9	0.92 (0.22–3.81)	0.905
	>9	0.50 (0.11–2.35)	0.378

Parity	Primiparous	Ref.	—
	Multiparous	1.22 (0.52–2.83)	0.649

Insemination time	Within 6 h	Ref.	
	Within 6–11 h	0.38 (0.21–0.71)	0.002
	>11 h	0.57 (0.11–2.90)	0.502

Body condition	Medium	Ref.	—
	Moderate	1.60 (0.87–2.95)	0.128

Inseminators	<5 years of experience	Ref.	—
	5–8 years of experience	1.23 (0.62–2.47)	0.547
	>8 years of experience	1.34 (0.61–2.99)	0.463

**Table 3:** Additional expenses for management procedures in cows that failed to conceive at their first AI, incurred due to a larger number of days open.

Item	Additional costs per cow/day in cows that did not conceive by their first service AI
Nutrition	Cost of nutrition per cow/d: 150 ETB
Labor	Labor cost per cow/d: 50 ETB
Milk loss	Milk lost per cow/d: (12 liter*∗*20 ETB) = 240 ETB
Total	440 ETB

1 ETB = 0.03 USD during the study.

## Data Availability

The data can be acquired from the principal author upon reasonable request.
